# Supplementary material for: Trophic ecology in an anchialine cave: A stable isotope study
Source: PLoS One. 2025 Jan 3;20(1):e0315821. doi: 10.1371/journal.pone.0315821 (PMC11698404; doi:10.1371/journal.pone.0315821)

PLOS ONE

Trophic ecology in an anchialine cave: a stable isotope study

--Manuscript Draft--

**Manuscript Number:**	PONE-D-24-24608
**Article Type:**	Research Article
**Full Title:**	Trophic ecology in an anchialine cave: a stable isotope study
**Short Title:**	Trophic ecology in an anchialine cave
**Corresponding Author:**	Fernando Alvarez, Ph.D.<br>Universidad Nacional Autonoma de Mexico<br>Mexico, Ciudad de México MEXICO
**Keywords:**	δ13C, δ15N, Yucatan Peninsula, Flooded caves
**Abstract:**	The analysis of carbon and nitrogen stable isotopes (δ13C and δ15N) has been widely used in ecology since it allows to identify the circulation of energy in a trophic network. The anchialine ecosystem is one of the less explored aquatic ecosystems in the world and stable isotope analysis represents a useful tool to identify the routes through which energy flow and to define the trophic niches of species. Sampling and ~~data~~ recording was conducted in one anchialine cave, Cenote Vaca Ha, near the town of Tulum, Quintana Roo, Mexico, where seven stygobitic species unique to the anchialine caves of the Yucatan Peninsula, plus sediment, water and vegetation samples were analyzed. We compared our results with two previous studies, one conducted in the same cave and another one from a cave in the same area, both based on the same species. Our study revealed: a) that despite a certain amount of variation in the δ13C and δ15N values of the species through time, both seasonally and interannually, the anchialine niche is much conserved; b) through contribution models we propose what are the most probable food sources for the studied species and the results confirm previous classifications; and c) that the shrimp Typhlatya pearsei presents very negative δ13C values, suggesting their consumption of bacterial sources consistent with a chemosynthetic origin of organic matter. The implications of the new findings show a very stable ecosystem with the shrimp Typhlatya pearsei, as the key species to link chemosynthetic microbial production of organic matter to the anchialine trophic web.
**Order of Authors:**	Brenda Durán, M.Sc.
	Fernando Alvarez, Ph.D.
**Additional Information:**	
**Question**	**Response**
**Financial Disclosure**<br><br>Enter a financial disclosure statement that describes the sources of funding for the work included in this submission. Review the submission guidelines for detailed requirements. View published research articles from *PLOS ONE* for specific examples.<br><br>This statement is required for submission and **will appear in the published article** if the submission is accepted. Please make sure it is accurate.	Yes

Powered by Editorial Manager® and ProduXion Manager® from Aries Systems Corporation

**Funded studies**<br>Enter a statement with the following details:<br>• Initials of the authors who received each award<br>• Grant numbers awarded to each author<br>• The full name of each funder<br>• URL of each funder website<br>• Did the sponsors or funders play any role in the study design, data collection and analysis, decision to publish, or preparation of the manuscript?<br><br>Did you receive funding for this work?	
Please add funding details.<br>as follow-up to "**Financial Disclosure**<br><br>Enter a financial disclosure statement that describes the sources of funding for the work included in this submission. Review the submission guidelines for detailed requirements. View published research articles from *PLOS ONE* for specific examples.<br><br>This statement is required for submission and **will appear in the published article** if the submission is accepted. Please make sure it is accurate.<br><br>**Funded studies**<br>Enter a statement with the following details:<br>• Initials of the authors who received each award<br>• Grant numbers awarded to each author<br>• The full name of each funder<br>• URL of each funder website<br>• Did the sponsors or funders play any role in the study design, data collection and analysis, decision to publish, or preparation of the manuscript?<br><br>Did you receive funding for this work?"	BD CONAHCYT graduate scholarship<br>FA grant PAPIIT IN206523 (DGAPA-UNAM)<br>FA grant CONAHCYT Ciencia Básica A1-S-32846
Please select the country of your main research funder (please select carefully as in some cases this is used in fee calculation).	MEXICO - MX

as follow-up to "**Financial Disclosure**<br><br>Enter a financial disclosure statement that describes the sources of funding for the work included in this submission. Review the submission guidelines for detailed requirements. View published research articles from *PLOS ONE* for specific examples.<br><br>This statement is required for submission and **will appear in the published article** if the submission is accepted. Please make sure it is accurate.<br><br>**Funded studies**<br>Enter a statement with the following details:<br>• Initials of the authors who received each award<br>• Grant numbers awarded to each author<br>• The full name of each funder<br>• URL of each funder website<br>• Did the sponsors or funders play any role in the study design, data collection and analysis, decision to publish, or preparation of the manuscript?<br><br>Did you receive funding for this work?"	
**Competing Interests**<br><br>Use the instructions below to enter a competing interest statement for this submission. On behalf of all authors, disclose any competing interests that could be perceived to bias this work—acknowledging all financial support and any other relevant financial or non-financial competing interests.<br><br>This statement is required for submission and **will appear in the published article** if the submission is accepted. Please make sure it is accurate and that any funding sources listed in your Funding Information later in the submission form are also declared in your Financial Disclosure statement.	The authors have declared that no competing interests exist.

View published research articles from *PLOS ONE* for specific examples.<br><br>**NO authors have competing interests**<br><br>Enter: *The authors have declared that no competing interests exist.*<br><br>**Authors with competing interests**<br><br>Enter competing interest details beginning with this statement:<br><br>*I have read the journal's policy and the authors of this manuscript have the following competing interests: [insert competing interests here]*<br><br>* typeset	
**Ethics Statement**<br><br>Enter an ethics statement for this submission. This statement is required if the study involved:<br><br>• Human participants<br>• Human specimens or tissue<br>• Vertebrate animals or cephalopods<br>• Vertebrate embryos or tissues<br>• Field research<br><br>Write "N/A" if the submission does not require an ethics statement.<br><br>General guidance is provided below. Consult the submission guidelines for detailed instructions. **Make sure that all information entered here is included in the Methods section of the manuscript.**	Collection of organisms was conducted under permit SAGARPA PPF/DGOPA-084/22 awarded to FA

**Format for specific study types**<br><br>**Human Subject Research (involving human participants and/or tissue)**<br>• Give the name of the institutional review board or ethics committee that approved the study<br>• Include the approval number and/or a statement indicating approval of this research<br>• Indicate the form of consent obtained (written/oral) or the reason that consent was not obtained (e.g. the data were analyzed anonymously)<br><br>**Animal Research (involving vertebrate animals, embryos or tissues)**<br>• Provide the name of the Institutional Animal Care and Use Committee (IACUC) or other relevant ethics board that reviewed the study protocol, and indicate whether they approved this research or granted a formal waiver of ethical approval<br>• Include an approval number if one was obtained<br>• If the study involved *non-human primates*, add *additional details* about animal welfare and steps taken to ameliorate suffering<br>• If anesthesia, euthanasia, or any kind of animal sacrifice is part of the study, include briefly which substances and/or methods were applied<br><br>**Field Research**<br><br>Include the following details if this study involves the collection of plant, animal, or other materials from a natural setting:<br>• Field permit number<br>• Name of the institution or relevant body that granted permission	
**Data Availability**<br><br>Authors are required to make all data underlying the findings described fully available, without restriction, and from the time of publication. PLOS allows rare exceptions to address legal and ethical concerns. See the PLOS Data Policy and FAQ for detailed information.	Yes - all data are fully available without restriction

Powered by Editorial Manager® and ProduXion Manager® from Aries Systems Corporation

A Data Availability Statement describing where the data can be found is required at submission. Your answers to this question constitute the Data Availability Statement and **will be published in the article**, if accepted.<br><br>**Important:** Stating 'data available on request from the author' is not sufficient. If your data are only available upon request, select 'No' for the first question and explain your exceptional situation in the text box.<br><br>Do the authors confirm that all data underlying the findings described in their manuscript are fully available without restriction?	
**Describe where the data may be found in full sentences. If you are copying our sample text, replace any instances of XXX with the appropriate details.**<br><br>• If the data are **held or will be held in a public repository**, include URLs, accession numbers or DOIs. If this information will only be available after acceptance, indicate this by ticking the box below. For example: *All XXX files are available from the XXX database (accession number(s) XXX, XXX.).*<br>• If the data are all contained **within the manuscript and/or Supporting Information files**, enter the following: *All relevant data are within the manuscript and its Supporting Information files.*<br>• If neither of these applies but you are able to provide **details of access elsewhere**, with or without limitations, please do so. For example:<br><br>*Data cannot be shared publicly because of [XXX]. Data are available from the XXX Institutional Data Access / Ethics Committee (contact via XXX) for researchers who meet the criteria for access to confidential data.*<br><br>*The data underlying the results presented in the study are available from (include the name of the third party*	All relevant data are within the manuscript and its Supporting Information files.

and contact information or URL).<br>• This text is appropriate if the data are owned by a third party and authors do not have permission to share the data.<br><br>* typeset	
Additional data availability information:	

manuscript

Click here to access/download;Manuscript;Trophic ecology in an anchialine cave final..docx

Trophic ecology in an anchialine cave: a stable isotope study

Brenda Durán [1, 2] and Fernando Álvarez [2] *

[1] Posgrado en Ciencias Biológicas, Universidad Nacional Autónoma de México, Ciudad Universitaria, 04510 Ciudad de México, Mexico

[2] Colección Nacional de Crustáceos, Instituto de Biología, Universidad Nacional Autónoma de México, Ciudad Universitaria, 04510 Ciudad de México, Mexico

*Corresponding author

E-mail: falvarez@unam.mx

Abstract

The analysis of carbon and nitrogen stable isotopes ($\delta^{13}C$ and $\delta^{15}N$) has been widely used in ecology since it allows to identify the circulation of energy in a trophic network. The anchialine ecosystem is one of the less explored aquatic ecosystems in the world and stable isotope analysis represents a useful tool to identify the routes through which energy flow and to define the trophic niches of species. Sampling and data recording was conducted in one anchialine cave, Cenote Vaca Ha, near the town of Tulum, Quintana Roo, Mexico, where seven stygobitic species unique to the anchialine caves of the Yucatan Peninsula, plus sediment, water and vegetation samples were analyzed. We compared our results with two previous studies, one conducted in the same cave and another one from a cave in the same area, both based on the same species. Our study revealed: a) that despite a certain amount of variation in the $\delta^{13}C$ and $\delta^{15}N$ values of the species through time, both

seasonally and interannually, the anchialine niche is much conserved; b) through contribution models we propose what are the most probable food sources for the studied species and the results confirm previous classifications; and c) that the shrimp *Typhlatya pearsei* presents very negative $\delta^{13}C$ values, suggesting their consumption of bacterial sources consistent with a chemosynthetic origin of organic matter. The implications of the new findings show a very stable ecosystem with the shrimp *Typhlatya pearsei*, as the key species to link chemosynthetic microbial production of organic matter to the anchialine trophic web.

Keywords: $\delta^{13}C$, $\delta^{15}N$, Yucatan Peninsula, Flooded caves

Introduction

Stable isotope analysis (SIA) has significantly contributed to identify the major routes through which energy flow in different ecosystems and has been an important tool to define trophic structure and to visualize the number of trophic levels in a given ecosystem (Peterson & Fry, 1987; Vander Zanden & Rasmussen, 2001). In hard to access ecosystems where sampling is technically difficult, such as the flooded caves of the anchialine ecosystem, SIA offers a way to explore the structure of the trophic web through the analysis of samples of sediment, water, organisms, and the surrounding vegetation. The data obtained through this approach can be complemented with occasional and/or opportunistic observations, or through laboratory experiments (Chávez-Solís et al., 2018).

Anchialine ecosystems have a rich and endemic fauna around the world that typically thrive in low nutrient conditions (Iliffe & Álvarez, 2018). With a reduced rate of transport of nutrients into the flooded caves (Pacioglu et al., 2023), the recurrent question has been, how can a diverse fauna with some locally abundant species develop and survive

under these conditions? Specially in the Yucatan Peninsula (YP), where flooded caves can form networks spanning hundreds of kilometers (QRSS, 2023), the conditions and mechanisms that shape the trophic web operate at various scales. There could be a short distance nutrient input that is present in the cave area adjacent to openings, such as the cenote pool, or cracks that connect the exterior to the aquifer; however, in remote areas inside the caves, hundreds or even thousands of meters from an opening to the exterior, other mechanisms may be operating different from the direct entry of organic material from the exterior. Thus, identifying the main nutrient sources and the major paths through which energy flow becomes of primary importance. We based our study in a cenote and its associated flooded cave that is typical of the cave area that develops along the Caribbean coast of Mexico (Fig. 1).

Previous studies of the trophic structure of anchialine communities using SIA (e.g., Pohlman et al., 1997; Humphreys, 1999; Pohlman, 2011; Lojen et al., 2012; Neisch et al., 2012; Chávez-Solís et al., 2020) identified a common pattern where anchialine fauna have $\delta^{13}C$ and $\delta^{15}N$ values that reflect the influence of the vegetation surrounding the cave entrance, in the YP these dolines or exterior connections to the aquifer are known as "cenotes". Derived from these data short trophic webs have been described, with a first trophic level composed of filter feeders that consume particulate organic matter or grazers that feed on the biofilm and a second level composed of predatory species (Pohlman et al., 1997). In the caves of the YP the first trophic level includes species such as the mysid shrimp *Antromysis cenotensis* which typically occurs near the caves' entrances or even in the twilight zone and feeds on finely particulate organic matter (Liévano-Beltrán & Simões, 2021) or the shrimps of the genus *Typhlatya* which possess a feeding apparatus made up of specialized setae to graze on the bacterial mats (Trejo-Ventura et al., 2023). The species

that compose the second trophic level are opportunistic predators such as the remipedes of the genus *Xibalbanus*, the brotulid fish *Typhlias pearsei* or the palaemonid shrimp *Creaseria morleyi* which have been observed preying on *Typhlatya* shrimps (Chávez-Solís et al., 2018; pers. obs.). Another significant component of the trophic web in the anchialine caves of the YP is the isopod *Creaseriella anops*, one of the most abundant organisms in this ecosystem, which acts as a scavenger that rapidly consumes all dead animals and decaying debris (Durán & Álvarez, 2021).

Although the entry of organic matter to the flooded caves from the outside is evident, at least in passageways adjacent to connections to the exterior, in several instances direct observations and SIA have identified a second possible source of nutrients that involves the degradation of organic matter by nitrifying bacteria and, depending on the type of cave and available dissolved compounds, the use of methane by chemoautotrophic bacteria to produce organic matter (Pohlman et al., 1997; Humphreys, 1999). An important bacterial and archaea diversity has been identified in cenotes and caves of the YP with a clear zonation according to the distance to the coastline and thus the influence of saltwater penetration that creates different geochemical conditions (Suárez-Moo et al., 2022). The overall contribution of bacterial activity to the anchialine trophic web is becoming increasingly relevant as exploration advances and the enormous lengths of the caves in the Yucatan (in the hundreds of kilometers) pose doubts about the effectiveness of water circulation to spread nutrients throughout.

In a few instances, very negative $\delta^{13}C$ values (<-36‰) have been recorded in the YP caves obtained from crustaceans in general or shrimp that are consistent with their feeding on nitrifying bacteria (Pohlman, 2011; Neisch et al., 2012). Brankovits et al. (2017) building on these ideas showed that a chemosynthetic route can operate under particular

conditions inside anchialine caves, providing a possible explanation for the impoverished $\delta^{13}C$ values that have been obtained (Chávez-Solís et al., 2020). Pohlman et al. (1997) had hypothesized that one thermosbaenacean, *Tulumella unidens*, and the atyid shrimp *Typhlatya mitchelli*, could be feeding on sulfide-oxidizing and methanotrophic bacteria.

Recent studies using a variety of techniques have identified a chemosynthetic route through the degradation of methane by bacteria to produce organic matter in an anchialine cave near Tulum, Quintana Roo, Mexico (Brankovits et al., 2017, 2018). With these important contributions to the understanding of the anchialine trophic web, several critical questions arise. Firstly, considering that there are thousands of flooded caves in the YP, how widespread is this mechanism? That is, the very negative $\delta^{13}C$ values can be found in all caves in the YP? Secondly, what species are involved in this process and how their distribution can affect such biogeochemical process? Thirdly, does the presence of these processes influence the distribution of species creating, where they operate, hotspots of diversity?

Another relevant question regarding the stability of the anchialine trophic web is if it is influenced by seasonal (rainy vs. dry seasons) or interannual changes due, for example, to the large-scale impacts of deforestation or the occurrence of hurricanes. Since SIA reflects what organisms have incorporated in the previous weeks or months, it should be possible to detect these changes obtaining an idea of how the anchialine ecosystem reacts to them. In this study, we compare the $\delta^{13}C$ and $\delta^{15}N$ values of seven anchialine species, sediment and organic matter in one cave in two seasons (rainy and dry). To explore variation among years, we compare also the new results with the values of Pohlman et al. (1997), obtained from a cave very close to our study site and based on the same species, and from Durán (2020) form the same cave but obtained 2 years before.

Materials and methods

The study was conducted in Cenote Vaca Ha and its associated cave, near the town of Tulum in the State of Quintana Roo, Mexico. Cenote Vaca Ha (20º16'14" N, 87º28'49" W) is located 6.67 km from the coastline, the pool is completely exposed and has an area of ~25 m^2 (Fig 1). Cenote Vaca Ha is surrounded by vegetation, mostly grasses and perennial trees, some palm trees are found in the surrounding areas far from the cenote's water body, in the rainy season a large swamp area develops that connects to the cenote pool. The site is only used by divers. The cenote was named "Vaca Ha" (cow water) because the land where it was discovered was used as a livestock maintenance site, taking advantage of the cenote pool as a watering hole. Currently the land is private, and divers pay an entry fee. The interior of the cave is known for containing wide passages, enormous columns and a rich decoration of speleothems, the passage reaches a depth of 24 m where it is possible to observe the halocline.

Fig 1. Map of the Yucatan Peninsula, Mexico, with cenotes shown in blue circles, and Cenote Vaca Ha indicated by a red circle.

Despite the continuous exploration of flooded caves in the area, and the presence of two of the largest cave systems in the world (Ox Bel Ha and Sac Actun) at a very short distance (< 10 km), Cenote Vaca Ha and its associated cave remains as an unconnected independent system. Notwithstanding the absence of known connections, there is obvious water circulation inside the cave.

Field work

Sediment samples were collected in 50 ml Falcon tubes, water in collapsible 5-liter plastic bags, and stygobitic fauna individually in small vials. Collection of organisms was conducted under permit SAGARPA PPF/DGOPA-084/22. In addition, vegetation samples from the rainforest floor around the cenote were also collected with tweezers. All samples were placed in sterile plastic vials. Water column profiles were obtained with a YSI EXO 3 sonde programmed to take readings every 15 seconds of: depth (m), temperature (ºC), pH, conductivity (mSc), salinity (ppt) and dissolved oxygen concentration (mg/L).

Samplings were conducted by two cave divers in July 2022 for the rainy season and in March 2023 for the dry season. Every dive lasted about 1 h, in case some of the samples or data were missed, a second dive was done the next day to complete the sampling.

Sample processing for carbon content analysis in water and sediments

To estimate the total dissolved carbon, organic and inorganic, 100 ml water samples were collected in ambar vials after filtering using pretreated (ignited at 500 ºC for 4 h) glass fiber filters (0.7 µm). Water samples were taken in the cenote pool and in the cave, in the cave two types of samples were taken, one in the freshwater layer at 8 m depth and one in salt water at a depth of 23 m. These samples were analyzed at the Laboratorio Universitario de Nanotecnología Ambiental (LUNA), of the Instituto de Ciencias Aplicadas y Tecnología (ICAT), UNAM using a Total Organic Carbon Analyzer (Shimadzu TOC-L CSH/CSN).

To obtain the amount of total carbon (organic and inorganic) present in the sediment of the cenote pool and in the cave´s interior, samples were taken with Falcon tubes. Five cave sediment samples were taken, each one 15 m apart starting the first sample in the cenote pool (Fig 2). The samples were dried at 60 ºC for 48 h and analyzed at LUNA of ICAT-UNAM using the same Total Organic Carbon Analyzer as above but with the adapter for solid samples SSM-5000a.

Fig 2. Schematic representation of Cenote Vaca Ha showing the different sections of the system, the two main water masses and the sites where water samples were taken for carbon measurements.

Sample processing for SIA

Sediment samples were treated with hydrochloric acid (HCl) before they were analyzed to eliminate all carbonates following the technique mentioned by Harris et al. (2001). The sediment and vegetation samples were dried at 60 ºC for 48 h, and then grinded until a fine powder was obtained.

Seven species of crustaceans collected were maintained live in 500 ml plastic containers with filtered (0.45 µm) cave water and aeration for 24 h. During this period the organisms would empty their digestive tracts and water was changed once to avoid coprophagy (Reid et al., 2008). The organisms were then placed in 50 ml sterile vials and frozen at -4 °C; the samples were taken to the laboratory, defrosted at room temperature and dried at 60 ºC for 48 h, and then grinded until a fine powder was obtained.

All samples, placed in tin capsules, were analyzed in a mass spectrometer (Isotopic Ratio Spectrometer, Isoprime 100TM, CF-EA) at the Stable Isotopes Laboratory of the Geotop Center at UQAM (Université du Québec à Montréal, Montréal, Québec, Canada). Carbon and nitrogen isotope ratios are expressed as $\delta^{13}C$ to Vienna Pee Dee Belemnite standard and as $\delta^{15}N$ to air.

Data analysis

To determine if the season of the year has an effect on the $\delta^{13}C$ and $\delta^{15}N$ values, the means for every species, for the dry and rainy seasons were compared with a two-way ANOVA. Post-hoc comparisons (Tukey test) were done for each species.

To graphically represent the isotopic values of the proposed sources as well as those obtained in the tissue of the consumer species (average values and their standard deviation), we constructed Bayesian mixing models (BMMs) with the MixSIAR R package using $\delta^{13}C$ and $\delta^{15}N$ as isotopic tracers to estimate the proportional contribution of potential food sources to the their diets (MixSIAR, Bayesian mixing models in R; Stock et al., 2018), this package is included in the R software (R Development Core Team, 2005). We used the classic TDF 0.5‰ for $\delta^{13}C$ and 3.4‰ for $\delta^{15}N$. The models obtained estimate the proportional contribution of the proposed sources considering the values of the isotopic signal of the consumers and the existing isotopic fractionation. The results are presented as credibility intervals (50, 75 and 95) that describe the range of possible contributions from a particular source.

To explore how the isotopic niche of the studied species changes through time we used the package SIBER in R to create scatter diagrams of the carbon and nitrogen data and independent ellipses, representing the isotopic niche (Jackson et al., 2011), for each of the analyzed datasets: Pohlman et al. (1997), Durán (2020), and the rainy and dry seasons presented herein for 2022-2023. We used the standard ellipse area (SEA) method to compare between the analyzed groups which shows the 50, 75 and 95% credibility intervals.

Results

Physico-chemical characterization of the water column

The physico-chemical sonde profiles show a stratified water column with a well-defined halocline at around 21 m, overlapping values are due to the presence of a shallow dome inside the cave which is shallower than the bottom of the cenote's pool. At the

halocline salinity changed from 2 to 35 in less than a meter (Fig 3A). Dissolved oxygen concentration was close to 4 mg/L at the pool's surface, it dropped to less than 0.5 mg/L inside the cave's dome, was constant around 3 mg/L in the freshwater layer and then dropped again to less than 1 mg/L in the saltwater (Fig 3B). Temperature was around 25.9 ºC at the pool's surface, slightly decreasing to 25.7 at the pool's bottom, it increased about have a degree inside the cave´s dome and then remained constant down to the halocline where it slightly increased (Fig 3C). Finally, pH was about neutral throughout the freshwater layer, except in the cave´s dome where it slightly increased 3 tenths of a unit, it decreased within the halocline and recovered in the saltwater layer (Fig. 3D). In general, no significant variations are observed between the rainy and dry seasons in any of the four variables measured.

Fig 3. Physico-chemical profiles of the water column in Cenote Vaca Ha for the rainy and dry seasons.
(A) salinity, (B) dissolved oxygen concentration, (C) temperature, (D) pH.

Total dissolved carbon was highest in the cave's freshwater layer, decreasing slightly towards the cenote pool and decreasing sharply in the saltwater layer (Table 1). Organic carbon represented 90.5% of all the dissolved carbon, and both organic and inorganic carbon had the same behavior regarding concentration as the total organic carbon (Table 1). Total carbon in sediments decreased steadily along the transect that started in the jungle floor, through the cenote pool and inside the cave to a penetration of 75 m. The amount of total carbon decreased to 38.7% of what was available in the exterior and the organic and inorganic fractions varied inversely, with the former being more abundant in

the exterior and cenote pool, and the latter comprising almost all the available carbon inside the cave (Table 2).

Table 1. Dissolved carbon in three sections of the aquifer in Cenote Vaca Ha, the samples were of 100 ml. TC=total dissolved carbon, OC=dissolved organic carbon, IC=dissolved inorganic carbon.

	Cenote pool	Cave (fresh water)	Cave (salt water)
TC	81.82 mg/L	96.01 mg/L	41.53 mg/L
OC	74.04 mg/L	87.43 mg/L	36.46 mg/L
IC	7.78 mg/L	8.58 mg/L	5.07 mg/L

Table 2. Total carbon in sediments (organic and inorganic fraction) through a transect inside the aquifer, this transect begins at the cenote pool and is directed to the cave region. Sampling was carried out every 15 m until completing a total of 75 m in length. TC=total carbon in sediments, OC=organic carbon in sediments, IC= inorganic carbon in sediments.

	Total carbon in sediments (%)	Organic carbon in sediments (%)	Inorganic carbon in sediments (%)
Jungle floor	27.01	26.7	0.24
Cenote pool sediment	20.94	19.68	1.26
15 m	11.71	4.32	7.39
30 m	11.6	0.33	11.3

45 m	11.39	2.86	8.53
60 m	11.05	1.04	10.01
75 m	10.46	0.44	10.02

SIA analysis

Stable isotope values were obtained for seven anchialine species, all crustaceans, for the dry and rainy seasons. Among the species analyzed are primary consumers (atyid shrimps of the genus *Typhlatya*, the mysid *Antromysis cenotensis*), predators (the remipede *Xibalbanus tulumensis*, the amphipod *Tuluweckelia cernua*, the mysid *Stygiomysis cokei*) and a scavenger (the isopod *Creaseriella anops*). Six of the seven species had $\delta^{13}C$ values that ranged between -37.8±3.1132 for *Typhlatya mitchelli* in the rainy season, to -24.0±1.264 for *Creaseriella anops* also in the rainy season (Table 3). These values are consistent with the feeding on organic matter from the outside that enters the cave. The second atyid species *T. pearsei* had very negative $\delta^{13}C$ values (-53.2±1.9629 to -49.4±0.6) which can be explained by the consumption of organic matter derived from bacterial activity, specially methanotrophic bacteria (see Table 6). The $\delta^{15}N$ values for six of the seven species ranged from 8.1±1.4882 obtained for *Typhlatya mitchelli* in the rainy season, to 14.7±0.3345 for *Xibalbanus tulumensis* in the dry season (Table 3). Clearly separated from this group is *Typhlatya pearsei* with extremely low values of -0.3±0.2376 and 1.4±0.4825, for the rainy and dry seasons, respectively (Table 3, Fig 4).

Table 3. Mean values (± SD) of $\delta^{13}C$ and $\delta^{15}N$ of seven anchialine species collected in Cenote Vaca Ha, first value corresponds to the rainy season and the second one to the dry season.

Species	$\delta^{13}C$	$\delta^{15}N$	n
Antromysis cenotensis	-33.5±0.1234	13.0±1.1234	6
	-31.37±0.0587	11.2±0.0012	6
Stygiomysis cokei	-28.9±0.1565	13.5±0.0565	2
	-31.1±0.1	14.6±0.1873	4
Typhlatya mitchelli	-37.8±3.1132	8.1±1.4882	3
	-33.60±0.4	11.50±0.053	10
Typhlatya pearsei	-53.2±1.9629	-0.3±0.2376	2
	-49.4±0.6	1.4±0.4825	3
Creaseriella anops	-24.0±1.264	10.5±0.7758	2
	-32.8±1.2	8.2±0.4305	2
Tuluweckelia cernua	-30.4±0.9627	12.7±0.2157	3
	-30.6±0.2341	11.7±0.3468	4
Xibalbanus tulumensis	-35.9±0.3223	12.8±0.0234	2
	-30.9±0.1987	14.7±0.3345	2

Fig 4. Biplot of mean ± standard error of $\delta^{13}C$ and $\delta^{15}N$ values for four datasets: in green Pohlman et al. (1997), in blue Durán (2020), and Durán (2022) rainy (black) and dry (red) seasons. Shaded areas indicate the possible food source for the anchialine fauna.

The $\delta^{13}C$ values differed significantly among species, between the rainy and dry seasons and in the interaction of species by season (two-way ANOVA, Table 4). The post-hoc comparisons (Tukey test) per species show differences for four species: two primary consumers (*Typhlatya mitchelli*, *T. pearsei*), one predator (*Xibalbanus tulumensis*) and one scavenger (*Creaseriella anops*); whereas the species that had the same behavior in both seasons were one primary consumer (*Antromysis cenotensis*) and two predators (*Stygiomysis cokei*, *Tuluweckelia cernua*) (Table 5).

Table 4. Two-way Anova results for $\delta^{13}C$ comparison by species and season of the year in Cenote Vaca Ha.

	DF	Sum SQ	Mean SQ	F Value	Prob
Species	6	2,834.93	472.49	511.898	<2.2E-16 ***
Season	1	11.06	11.06	11.986	0.001245 **
Species/Season	6	300.84	50.14	54.322	<2.2E-16 ***
Residuals	42	38.77	0.92		

Table 5. Comparison of rainy and dry season $\delta^{13}C$ values per species, * denotes significant differences (Tukey test).

	p adj
Antromysis cenotensis	0.534
Creaseriella anops	0.000*

Stygiomysis cokei	0.131
Typhlatya mitchelli	0.000*
Xibalbanus tulumensis	0.002*
Typhlatya pearsei	0.000*
Tuluweckelia cernua	1.000

The Bayesian mixing model results show the relative contribution of the most likely food sources to the diet of six species (Fig 5, Table 6). The diet of the remipede *X. tulumensis* was largely based on the consumption of the mysid shrimp *A. cenotensis* and in a lesser proportion of the isopod *C. anops* and the atyid shrimp *T. mitchelli* in the rainy season (Fig 5A); whereas in the dry season its diet was almost completely composed of *T. mitchelli* (Fig 5H). The mysid shrimp *Stygiomysis cokei* also shows a change in the diet between seasons, it consumed about 70% of *A. cenotensis*, 25% *T. mitchelli*, and 5% of *C. anops* in the rainy season (Fig 5B), and about 95% the amphipod *T. cernua* and 5% *A. cenotensis* in the dry season (Fig 5I). The amphipod *Tuluweckelia cernua* shows a similar diet in both seasons consuming preferentially *T. mitchelli* and secondarily *C. anops* (Fig 5C, J). The diet of *T. mitchelli* is composed mainly of nitrifying bacteria in both seasons with a reduced contribution of particulate organic matter and cave sediment (Fig 5D, K). In the case of *T. pearsei*, with no seasonal variation, the feeding on methanotrophic bacteria could explain their low $\delta^{13}C$ values (Fig 5E, L). The diet of the mysid *A. cenotensis* is consistent with the consumption of nitrifying bacteria and particulate organic matter in both seasons (Fig 5F, M). Finally, the cirolanid *Creaseriella anops*, shows a preference for cave

sediment in the rainy season and a split preference for plant matter and the mysid *A. cenotensis* in the dry season (Fig 5G, N).

Fig 5. Bayesian mixing model results to estimate the most probable diet sources. (A, H) the remipede *Xibalbanus tulumensis*, (B, I) the mysid shrimp *Stygiomysis cokei*, (C, J) the amphipod *Tuluweckelia cernua,* (D, K) the atyid shrimp *Typhlatya mitchelli*, (E, L) the atyid shrimp *Typhlatya pearsei*, (F, M) the mysid shrimp *Antromysis cenotensis*, (G, N) the isopod *Creaseriella anops*. Abbreviations for the probable food sources are: AC, *Antromysis cenotensis*; CA, *Creaseriella anops*; TM, *Typhlatya mitchelli*; POM, particulate organic matter; NB, nitrifying bacteria; MB-BP, methanotrophic bacteria, data from Brankovits & Pohlman (2020); MB-WF, methanotrophic bacteria, data from Whiticar & Faber (1986).

Table 6. Mean $\delta^{13}C$ values ± standard deviation for additional possible sources of contribution to the diet of consumers in Cenote Vaca Ha. POM = particulate organic matter.

	Dry Season	Rainy Season
Source	$\delta^{13}C$	$\delta^{13}C$
POM in the cave	-22.23±0.1915	-22.419±0.5066
Cave sediment	-13.7564±0.3456	-27.6632±0.2916
Nitrifying bacteria	-39.8333±3.4302	-39.8333±3.4302
Methane oxidizing bacteria (Brankovits & Pohlman, 2020)	-64.3633±5.4242	-64.3633±5.4242

Methane oxidizing bacteria (Whiticar & Faber, 1986)	-68.3666±5.4271	-68.3666±5.4271
AC (*Antromysis cenotensis*)	-31.3157±1.2211	-32.8092±0.6088
CA (*Creaseriella anops*)	-32.8±1.2211	-24.0±1.2647
TM (*Typhlatya mitchelli*)	-33.60±0.4	-37.8±3.1132

To analyze the seasonal and interannual variation in the isotopic niche displayed by anchialine species we compared the results of this study with another three datasets using Bayesian ellipses (Fig 6). The four datasets show a considerable overlap (> 40% on average) for all species except for *T. pearsei* which due to very negative $\delta^{13}C$ values and very low $\delta^{15}N$ values lies outside the cluster formed by the rest of the anchialine species analyzed.

Fig 6. Comparison of the isotopic niche of anchialine species.
(A) Bayesian standard ellipses comparing the isotopic niche of anchialine species based on four data sets: Pohlman et al. (1997), Durán (2020), and Durán (2022) rainy and dry seasons. (B) density plots of Bayesian estimates of Standard Ellipse Areas (SEA) for the same datasets. Black dots indicate the modes of SEA, and boxed areas—Bayesian 50, 75 and 95% credible intervals; the red “x” indicate the mean.

The trophic level analysis was not conducted as no reliable values of $\delta^{15}N$ could be obtained from the particulate organic matter in the water column or from the sediment in

the cave, thus no primary production values were available to conduct the calculations. The N concentrations were below detection by the mass spectrometer.

Discussion

Environmental conditions

The water column conditions inside the cave were similar between the rainy and dry seasons showing little variation. The stratification remained without variation, with a distinct layer inside the dome of the cave, similar to what Brankovits et al. (2018) called a shallow halocline, which in our case shows changes in temperature, dissolved oxygen concentration and pH, but not in salinity; a homogeneous freshwater layer, from 5 to 21 m depth; and a well-defined halocline at 21-22 m depth. Dissolved carbon in the water column and total carbon in sediments showed a clear inverse pattern in the freshwater layer where organic carbon decreases advancing inside the cave and inorganic carbon increases, while small changes can be seen in the marine water layer due to the different dynamics that operate there. Although in the YP the aquifer in lowland areas close to the shoreline is influenced by high permeability of the substrate and thus high infiltration rates of meteoric water and the tidally influenced marine water intrusion below the freshwater lens, conditions are generally stable, but can reflect meteorological events such as strong storms or hurricanes with short-period peaks in water level and flow velocity (Gondwe et al., 2010; Brankovits et al., 2018).

Food web structure

Six of the seven anchialine species assessed from Cenote Vaca Ha form a well-defined cloud with $\delta^{13}C$ values that reflect the consumption of organic matter derived from the transport of organic matter from the rainforest floor. Both, the POM in the cave and the cave sediment have isotopic values consistent with the entering of organic matter from the

exterior. Several authors have obtained similar isotopic values form anchialine fauna in the YP (Chávez-Solís et al., 2020; Solís et al., 2022). Interestingly, the comparison of the four datasets considered, from 1993 through 1995 (Pohlman et al., 1997), 2019 (Durán, 2020) and this study including the 2022 rainy and 2023 dry seasons, that is a span of 30 years, shows a substantial overlap of the anchialine species isotopic niches. Temporal variation in isotopic niches has been widely documented at various scales, from weekly intervals to multi-year periods (Mittermayr et al., 2014). Being that stable isotopes reflect the organism's diet in relatively short periods of time (Kurle, 2009) it is remarkable that the anchialine animal community in two nearby caves, Cenote Mayan Blue (Pohlman et al., 1997) and Cenote Vaca Ha (Durán (2020) and this study) show such a reduced amount of variation through time. Some species, like the shrimp *T. mitchelli*, a grazer and primary consumer, remained within a narrow range of variation in both isotopic proportions (-33.0 to -37.8 δ^{13}C, 8.1 - 11.5 δ^{15}N) through time, whereas others, such as the predatory palaemonid shrimp *Creaseria morleyi* showed a wider range of variation (-25.13 to -36.4 δ^{13}C, 9.24 - 11.7 δ^{15}N).

The case of the atyid shrimp *Typhlatya pearsei*, which has very negative δ^{13}C values (-49.4 to -53.2‰), is interesting as these results place the species separate from the main isotopic space where the rest of the species overlap (Fig. 6). Clearly, *T. pearsei* has a different diet, possibly composed, at least partially, of methanotrophic bacteria (Fig. 5E, K), than those of the rest of the anchialine species which could be related to a spatial segregation or a different feeding behavior. In contrast to *T. mitchelli*, *T. pearsei* occurs in more internal sections of the cave where nutrient input from the exterior is limited and consequently could be consuming a biofilm composed by methanotrophic bacteria which have extremely low δ^{13}C values (Brankovits & Pohlman, 2020; Whiticar & Faber, 2020).

This scenario is consistent with the chemoautotrophic route proposed by Brankovits et al. (2017) described from the nearby Ox Bel Ha cave system. Although *Typhlatya* shrimp, due to their filtering/grazing feeding mode, were the key link assumed to be responsible for the introduction of chemoautotrophically derived organic matter into the food web, the question remained as to which one of the three possible species of *Typhlatya* was involved in this process. Chávez-Solís et al. (2020) recorded low $\delta^{13}C$ values for *T. pearsei* (-35.0 ± 1 to -41 ± 1‰) and *T. dzilamensis* (-31.0 ± 1 to -44 ± 0.6‰) but from different caves and times, thus lacking a context to compare these values. The values that Chávez-Solís et al. (2020) obtained for *T. dzilamensis*, which generally occurs below the halocline, were not explained fully since the methane concentrations as well as methanotrophic bacteria are present preferentially in the freshwater lens (Brankovits et al., 2017).

Although any of the three possible species of *Typhlatya* present in the area could be feeding on methanotrophic bacteria, since they all have similar feeding apparatuses (Trejo-Ventura et al., 2023), here we propose, based on our findings and previously reported values of $\delta^{13}C$ for *T. mitchelli,* that it is *T. pearsei* the key species that links the microbial processing of methane with the rest of the anchialine food web. *Typhlatya pearsei* is relatively rare, despite its wide distribution throughout the YP it always occurs in reduced numbers. In contrast, *T. mitchelli* is very common occurring from the surface to ~18 m depths in most caves that have been explored (Benítez et al., 2019). The rareness of *T. pearsei* could be very significant if it in fact represents one of the necessary components for the chemosynthesis-based food web to operate. The other necessary conditions are: rainfall, a well preserved rainforest on the surface to produce the organic matter that will decompose in the carbonate rock matrix, a cave conformation that allows for the formation of an anoxic freshwater layer next to the cave´s ceiling with methanogenic bacteria, the presence of

methanotrophic bacteria in a contiguous oxygenic layer that will use methane to synthesize organic matter, and the presence of *Typhlatya* shrimp to feed on this microbial mat introducing new organic matter into the food web. As we pointed out, the shrimp responsible for the last crucial step is *Typhlatya pearsei*, at least in the Caribbean cave area of Mexico as defined by Álvarez et al. (2023).

The tropic level of species was not determined since no reliable δ^{15}N base values could be obtained. Typically, δ^{15}N of the particulate organic matter in the water column or from the sediment is used as the base value, where bacteria, protists, algae, decaying plant material, and other small animals constitute a proxy to what could be considered the product of primary production. However, in this case N levels were too low to be detected. Nitrogen sources can be hard to identify as they depend on complex biogeochemical processes, microbial activity, amount of rainfall, and other direct sources (Townsend-Small et al., 2007; Reuss et al., 2013) and individuals of the same population may even show significant variation (Beaudoin et al., 1999). In this case the trophic roles of the studied species can be inferred from their morphology with support from observations in the field. *Typhlatya* shrimp have modified pereopods to filter feed or graze on the biofilm (Trejo-Ventura et al., 2023); the mysid shrimp *Antromysis cenotensis* is usually seen at shallow depths or near the surface feeding on small POM particles; the remipede *Xibalbanus tulumensis* is a carnivore, it was recognized as the first venomous crustacean in the world (von Reumont et al., 2014); the palaemonid shrimp *Creaseria morleyi* is also a predator althouh it can be an opportunistic omnivore (Chávez-Solís et al., 2018); the cirolanid isopod *Creaseriella anops* is a scavenger (Durán & Álvarez, 2021); and both the amphipod *Tuluweckelia cernua* and the mysid shrimp *Stygiomysis cokei* due to their size and type of

buccal appendages are clearly carnivores. Thus, we can infer the trophic role of these species based on their morpho-functional characteristics and $\delta^{13}C$ signatures.

Seasonal variation in isotopic niche

SIA has been used to determine changes through time in the diet or trophic position since organisms incorporate, in relatively short periods of time, the isotopic signature of their food items (e.g., Kozak et al., 2020; Quillfeldt & Masello, 2020). In various instances differences in isotopic values are the result of migrations or marked changes in the physical conditions of the habitat. We observed significant differences in the $\delta^{13}C$ values between the rainy and dry seasons in four out of the seven species analyzed. Water movement inside caves can be significant as freshwater slides towards the sea on top of a saline water intrusion that penetrates inland. Seasonal changes due to rainfall or strong tides create a very dynamic system specially in open conduits, as caves are, and more so in areas near the coastline (Beddows et al., 2007). Changes in salinity, temperature, conductivity, water level and other variables that describe the water column have been recorded in several sites along the Caribbean cave area (e.g., Sánchez et al., 2002; Gondwe et al., 2010; Brankovits et al., 2018). However, despite the variations in the physical environment anchialine species seem, through time, to be feeding on the same sources producing similar isotopic signatures.

The small variations observed in this study of the isotopic values suggest the stability of the anchialine ecosystem at least in terms of nutrient input. Brankovits et al. (2018) reported seasonal changes in the hydrological conditions of a cave due to rainfall in the same area as that of our study site and proposed that nutrient concentrations would decrease after strong rainfall due to a higher flow that would disperse them. So even if

fluctuations in nutrient concentrations are weather-driven, their origin and composition remain relatively stable.

The obtained results show an anchialine ecosystem that depends on the nutrients generated by the rainforest that develops on the surface influence area of the cave. Nutrients can enter via POM that collects in the cenote pool or through bacterial activity that transforms a variety of compounds into nutrients. In either case, the decaying organic matter from the rainforest is the primary source of nutrients for the anchialine species. Our results also show that the isotopic niches have only small variations with a substantial overlap of the community's isotopic space over time. In a sense, the predictability of the trophic behavior of the anchialine community renders it a very vulnerable one that could collapse as the surface environment is modified. We also foresee that the disappearance of key species, such as the shrimps of the genus *Typhlatya*, could draw with them complete microbial loops that are unique to this ecosystem, including the described chemosynthetic processing of methane. Refinements on the description of the anchialine trophic web are forthcoming with the use of modern sampling and analysis techniques.

Acknowledgements

The first author thanks the Posgrado en Ciencias Biológicas, UNAM and CONAHCYT for the graduate scholarship awarded. We gratefully acknowledge the funding received through grants PAPIIT IN206523 (DGAPA-UNAM) and CONAHCYT Ciencia Básica A1-S-32846. Field work was possible thanks to the help and assistance of O. Cortés, A. Mora, A. Ceballos, B. Espinosa, M. Vázquez, J.L. Villalobos and P. Kovac. We thank D. Planas, A. Adamowics and J.F. Hélie for their assistance during the stable isotope analysis at the Centre de recherche Geotop, Université du Québec à Montréal. We

thank J.C. Durán for his help with the elemental analyses of carbon and nitrogen in sediment and water at the Institute of Applied Science and Technology, UNAM.

References

Álvarez F, Durán B, Meacham S. Chapter 13. Anchialine fauna of the Yucatan Peninsula: diversity and conservation challenges. In: Jones RW, Ornelas-García CP, Pineda-López R, Álvarez F, editors. Mexican fauna in the Anthropocene. Cham, Switzerland: Springer Nature Switzerland AG; 2023. pp. 287-301. https://doi.org/10.1007/978-3-031-17277-9_13

Beaudoin CP, Tonn WM, Prepas EE, Wasenaar LI. Individual specialization and trophic adaptability of northern pike (*Esox lucius*): an isotope and dietary analysis. Oecologia. 1999;120: 386-396. https://doi.org/10.1007/s004420050871

Beddows PA, Smart PL, Whitaker FF, Smith SL. Decoupled fres-saline groundwater circulation of a coastal carbonate aquifer: spatial patterns of temperature and specific electrical conductivity. J Hydrol. 2007;346: 18-32. https://doi.org/10.1016/j.jhydrol.2007.08.013

Benítez S, Iliffe TM, Quiroz-Martínez B, Alvarez F. How is the anchialine fauna distributed within a cave? A study of the Ox Bel Ha System, Yucatan Peninsula, Mexico. Subt Biol. 2019;31: 15-28. https://doi.org/10.3897/subtbiol.31.34347

Brankovits D, Pohlman JW. Methane oxidation dynamics in a karst subterranean estuary. Geochim Cosmochim Ac. 2020;277: 320-333. https://doi.org/10.1016/j.gca.2020.03.007

Brankovits D, Pohlman JW, Niemann H, Leigh MB, Leewis MC, Becker KW, Iliffe TM, Alvarez F, Lehmann MF, Phillips B. Methane-and dissolved organic carbon-fueled

microbial loop supports a tropical subterranean estuary ecosystem. Nat Commun. 2017;8: 1835. https://doi.org/10.1038/s41467-017-01776-x

Brankovits D, Pohlman JW, Ganju NK, Iliffe TM, Lowell N, Roth E, Sylva SP, Emmert JA, Lapham LL. Hydrologic controls of methane dynamics in karst subterranean estuaries. Glob Biogeochem Cycles. 2018;32: 1759-1775. https://doi.org/10.1029/2018GB006026

Chávez-Solís EM, Mejía-Ortiz LM, Simões N. Predatory behavior of the cave shrimp *Creaseria morleyi* (Creaser, 1936) (Caridea: Palaemonidae), the blind hunter of the Yucatán cenotes, Mexico. J. Crust Biol. 2018;38: 1-7. https://doi.org/10.1093/jcbiol/rux098

Chávez-Solís EM, Solís C, Simões N, Mascaró M. Distribution patterns, carbon sources and niche partitioning in cave shrimps (Atyidae: *Typhlatya*). Sci Rep. 2020;10: 12812. https://doi.org/10.1038/s41598-020-69562-2

Durán B. Redes tróficas en los sistemas anquihalinos: un estudio de isotopía estable. M.Sc. Thesis, Universidad Nacional Autónoma de México. 2020. Available from: https://tesiunam.dgb.unam.mx/F/M94JUPLHNH4YND3P38BVXKUBAVC64F78DGT3BA9DSH4AFA79GI-26576?func=full-set-set&set_number=028127&set_entry=000002&format=999

Durán B, Álvarez F. La cochinilla *Creaseriella anops* (Crustacea: Isopoda: Cirolanidae) de las cuevas anquihalinas de Yucatán, México. ¿Existen patrones de variación en la talla? Rev Mex Biodivers. 2021;92: e923925. https://doi.org/10.22201/ib.20078706e.2021.92.3925

Gondwe BRN, Lerer S, Stisen S, Marín L, Rebolledo-Vieyra M, Merediz-Alonso G, Bauer-Gottwein P. Hydrogeology of the south-eastern Yucatan Peninsula: new insights

from water level measurements, geochemistry, geophysics and remote sensing. J Hydrol. 2010;389: 1-17. https://doi.org/10.1016/j.jhydrol.2010.04.044

Harris D, Horwath WR, van Kessel C. Acid fumigation of soils to remove carbonates prior to total organic carbon or carbon-13 isotopic analysis. Soil Sci Soc Am J. 2001;65: 1853-1856. https://doi.org/10.2136/sssaj2001.1853

Humphreys WF. Physico-chemical profile and energy fixation in Bundera Sinkhole, an anchialine remipede habitat in north-western Australia. J R Soc West Aust. 1999;82: 89-98.

Iliffe TM, Álvarez F. Chapter 18: Research in anchialine caves. In: Moldovan O, Kovác L, Halse S, editors. Cave Ecology. Cham, Switzerland: Springer; 2018, pp. 383-397. https://doi.org/10.1007/978-3-319-98852-8_18

Jackson AL, Parnell AC, Inger R, Bearhop S. Comparing isotopic niche widths among and within communities: SIBER - Stable Isotope Bayesian Ellipses in R. J Anim Ecol. 2011;80: 595-602. https://doi.org/10.1111/j.1365-2656.2011.01806.x

Kozak ER, Franco-Gordo C, Godínez-Domínguez E, Suárez-Morales E, Ambriz-Arreola I. Seasonal variability of stable isotope values and niche size in tropical calanoid copepods and zooplankton size fractions. Mar Biol. 2020;167: 37. https://doi.org/10.1007/s00227-020-3653-7

Kurle CM. Interpreting temporal variation in omnivore foraging ecology via stable isotope modelling. Funct Ecol. 2009;23: 733-744. https://doi.org/10.1111/j.1365-2435.2009.01553.x

Liévano-Beltrán LA, Simões N. Updated distribution of the mysid *Antromysis cenotensis* (Crustacea: Peracarida), a protected key species in Yucatan Peninsula cenotes. Diversity. 2021;13: 154. https://doi.org/10.3390/d13040154

Lojen S, Cukrov M, Cukrov N. Stable isotope composition of the serpulid *Ficopomatus enigmaticus* as environmental proxy. Nat Croat. 2012;21: 74-76.

Mittermayr A, Fox SE, Sommer U. Temporal variation in stable isotope composition ($\delta^{13}C$, $\delta^{15}N$ and $\delta^{34}S$) of a temperate *Zostera marina* food web. Mar Ecol Progr Ser. 2014;505: 95-105. https://doi.org/10.3354/meps10797

Neisch JA, Pohlman JW, Iliffe TM. The use of stable and radiocarbon isotopes as a method for delineating sources of organic material in anchialine systems. Nat Croat. 2012;21: 83-85.

Pacioglu O, Tusa IM, Popa I, Itcus C, Plavan GI, Boufahja F, Baba SC. Aquatic subterranean food webs: A review. Glob Ecol Conserv. 2023;48: e02704. https://doi.org/10.1016/j.gecco.2023.e02704

Peterson BJ, Fry B. Stable isotopes in ecosystem studies. Ann Rev Ecol Syst. 1987;18: 293-320. https://doi.org/10.1146/annurev.es.18.110187.001453

Pohlman JW. The biogeochemistry of anchialine caves: progress and possibilities. Hydrobiologia. 2011;677, 33-51. https://doi.org/10.1007/s10750-011-0624-5

Pohlman JW, Iliffe TM, Cifuentes LA. A stable isotope study of organic cycling and the ecology of an anchialine cave ecosystem. Mar Ecol Progr Ser. 1997;155: 17-27. https://doi.org/10.3354/meps155017

QRSS (Quintana Roo Speleological Survey). 2023. https://legacy.caves.org/project/qrss/qrlongesp.htm

Quillfeldt P, Masello JF. Compound-specific stable isotope analyses in Falkland Islands seabirds reveal seasonal changes in trophic positions. BMC Ecol. 2020;20: 21. https://doi.org/10.1186/s12898-020-00288-5

R Core Team (2020). R: A language and environment for statistical computing. R Foundation for Statistical Computing, Vienna, Austria. URL https://www.R-project.org/.

Reid DJ, Quinn GP, Lake PS, Reich P. Terrestrial detritus supports the food webs in lowland intermittent streams of south-eastern Australia: a stable isotope study. Fresh Biol. 2008;53: 2036-2050. https://doi.org/10.1111/j.1365-2427.2008.02025.x

Reuss NS, Hamerlik L, Velle G, Michelsen A, Pedersen O, Brodersen KP. Stable isotopes reveal that chironomids occupy several trophic levels within West Greenland lakes: Implications for food web studies. Limnl Ocean. 2013;58: 1023-1034. https://doi.org/10.43.19/lo.2013.58.3.1023

Sánchez M, Alcocer J, Escobar E, Lugo A. Phytoplankton of cenotes and anchialine caves along a distance gradient from the northeastern coast of Quintana Roo, Yucatan Peninsula. Hydrobiologia. 2002;467: 79-89. https://doi.org/10.1023/A:1014936714964

Solís C, Chávez-Solís EM, Rodríguez-Ceja, M, Méndez-García CG, Ortíz E, Canto C, Martínez-Carrillo MA, Mascaró M. Linking radiocarbon and trophic webs in karstic groundwater ecosystems in the Yucatan Peninsula, Mexico. Radiocarbon. 2022;64: 1629-1639. https://doi.org/10.1017/RDC.2022.100

Stock BC, Jackson AL, Ward EJ, Parnell AC, Phillips DL, Semmens BX. Analyzing mixing systems using a new generation of Bayesian tracer mixing models. PeerJ. 2018;6: e5096. https://doi.org/10.7717/peerj.5096.

Suárez-Moo P, Remes-Rodríguez CA, Márquez-Velázquez NA, Falcón LI, García-Maldonado JQ, Prieto-Davó A. Changes in the sediment microbial community

structure of coastal and inland sinkholes of a karst ecosystem from the Yucatan peninsula. Sci Rep. 2022;12: 1110. https://doi.org/10.1038/s41598-022-05135-9

Townsend-Small A, McCarthy MJ, Brandes JA, Yang L, Zhang L, Gardner WS. (2007). Stable isotopic composition of nitrate in Lake Taihu, China, and major inflow rivers. In: Qin B, Liu Z, Havens K, editors. Eutrophication of Shallow Lakes with Special Reference to Lake Taihu, China. Developments in Hydrobiology, vol 194. Springer, Dordrecht. https://doi.org/10.1007/978-1-4020-6158-5_15

Trejo-Ventura I, Armendáriz G, Villalobos JL, Álvarez F. Morphology of the feeding apparatus of cave shrimps of the genus *Typhlatya* (Decapoda: Atyidae) from the Yucatán Peninsula and comparison with epigean species. Rev Mex Biodivers. 2023;94: e945230. https://doi.org/10.22201/ib.20078706e.2023.94.5230

Vander Zanden MJ, Rasmussen JB. Variation in $\delta^{15}N$ and $\delta^{13}C$ trophic fractionation: implications for aquatic food web studies. Limnol Oceanogr. 2001;46: 2061-2066. https://doi.org/10.4319/lo.2001.46.8.2061

von Reumont BM, Blanke A, Richter S, Alvarez F, Bleidorn C, Jenner RA. The first venomous crustacean revealed by transcriptomics and functional morphology: remipede venom glands express a unique toxin cocktail dominated by enzymes and a neurotoxin. Mol Biol Evol. 2014;31: 48-58. https://doi.org/10.1093/molbev/mst199

Whiticar MJ, Faber E. Methane oxidation in sediment and water column environments-Isotope evidence. Org Geochem. 1986;10: 759-768. https://doi.org/10.1016/S0146-6380(86)80013-4

Figure 1 Click here to access/download;Figure;Fig 1.tif

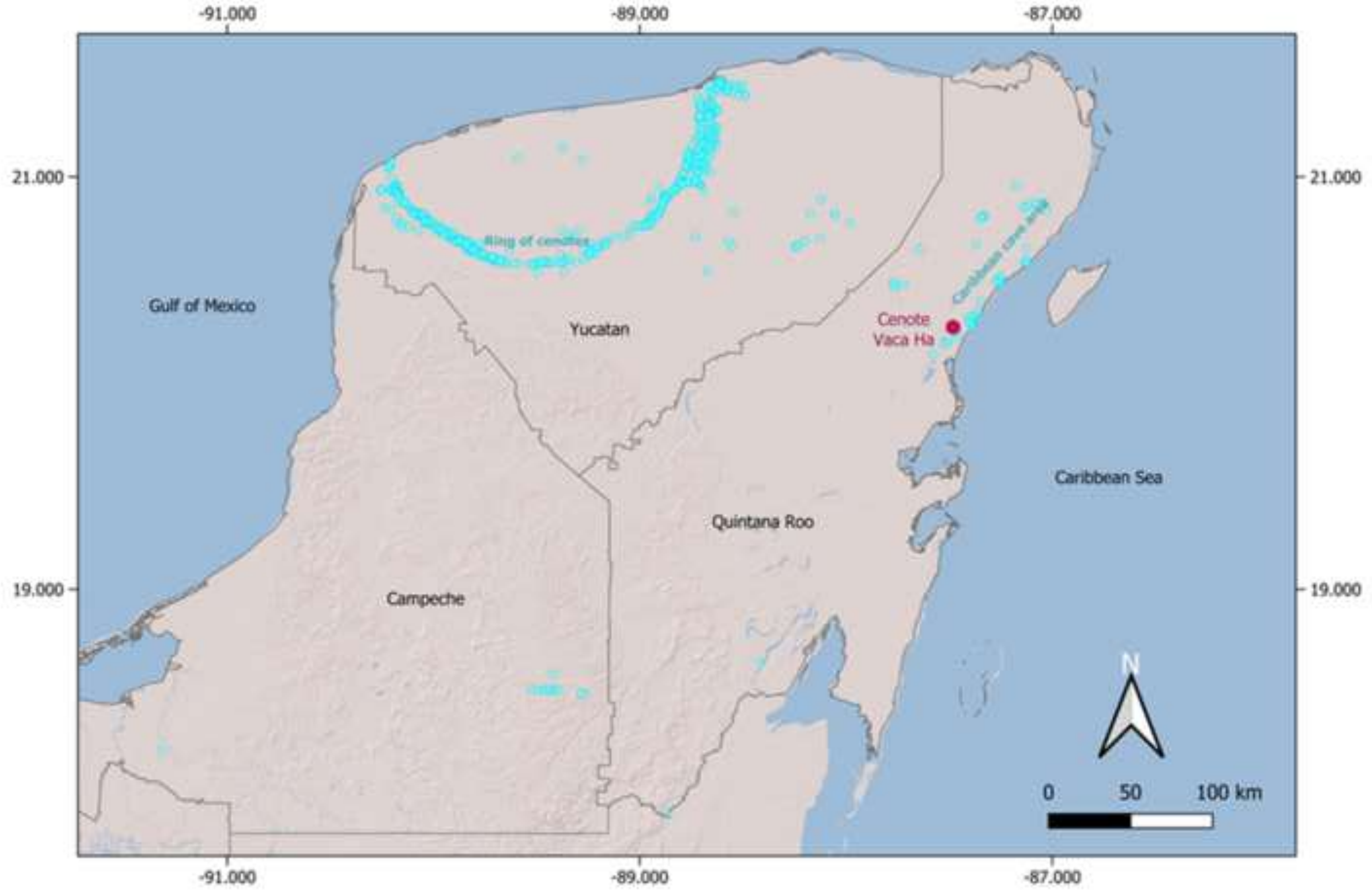

Figure 2

Click here to access/download;Figure;Fig 2.tif

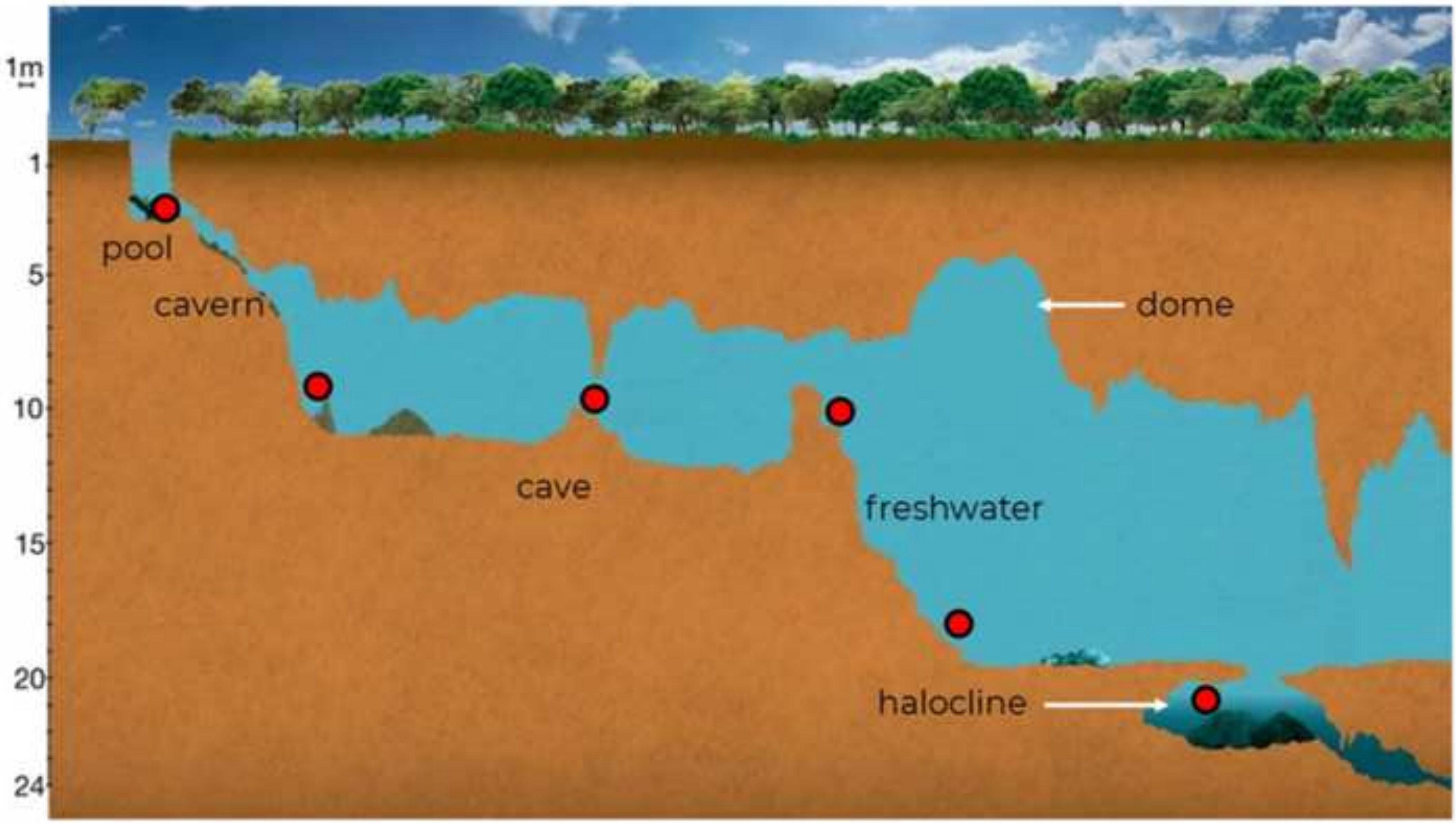

Figure 3

Click here to access/download;Figure;Fig 3.tif

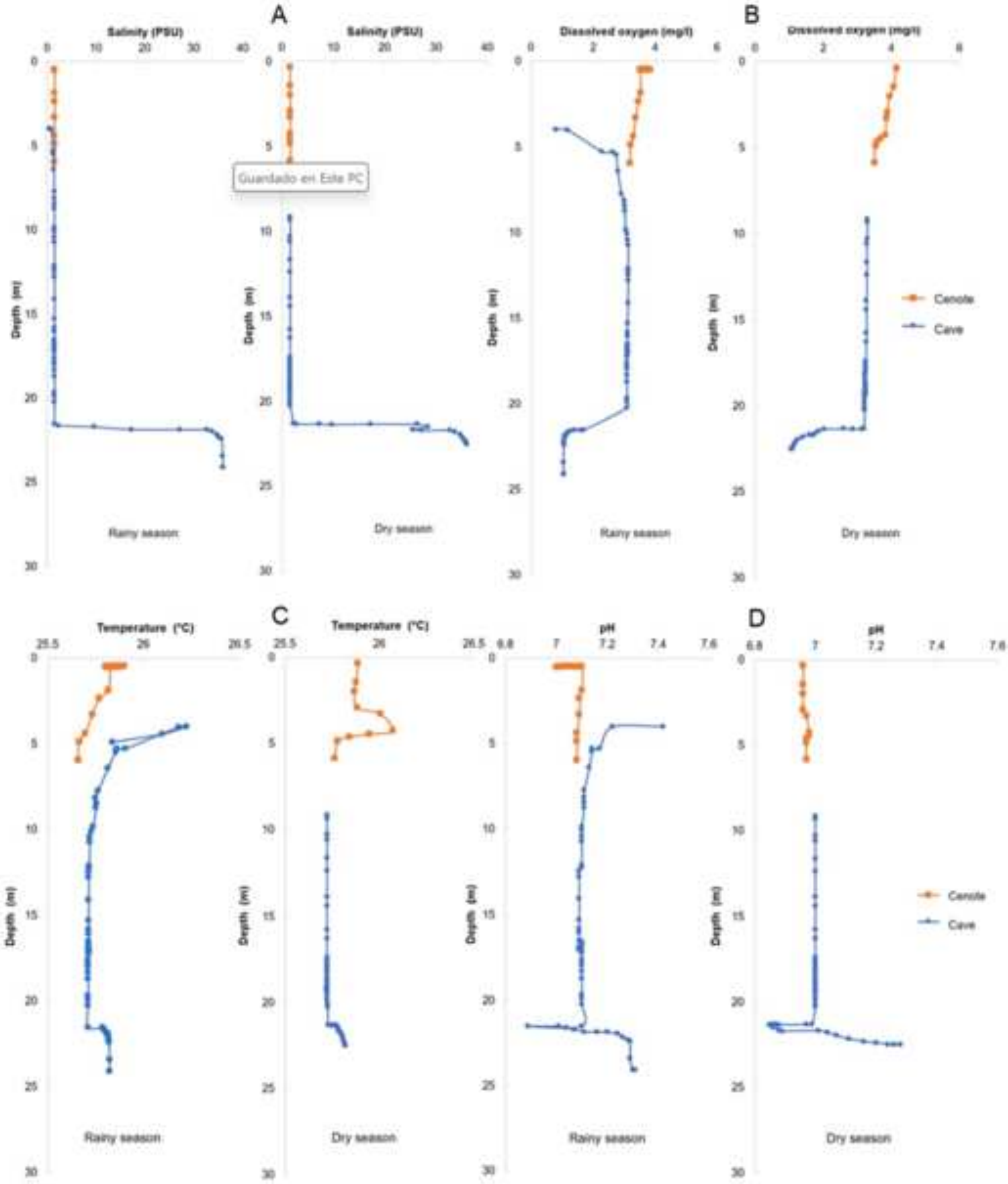

Figure 4

Click here to access/download;Figure;Fig 4.tif

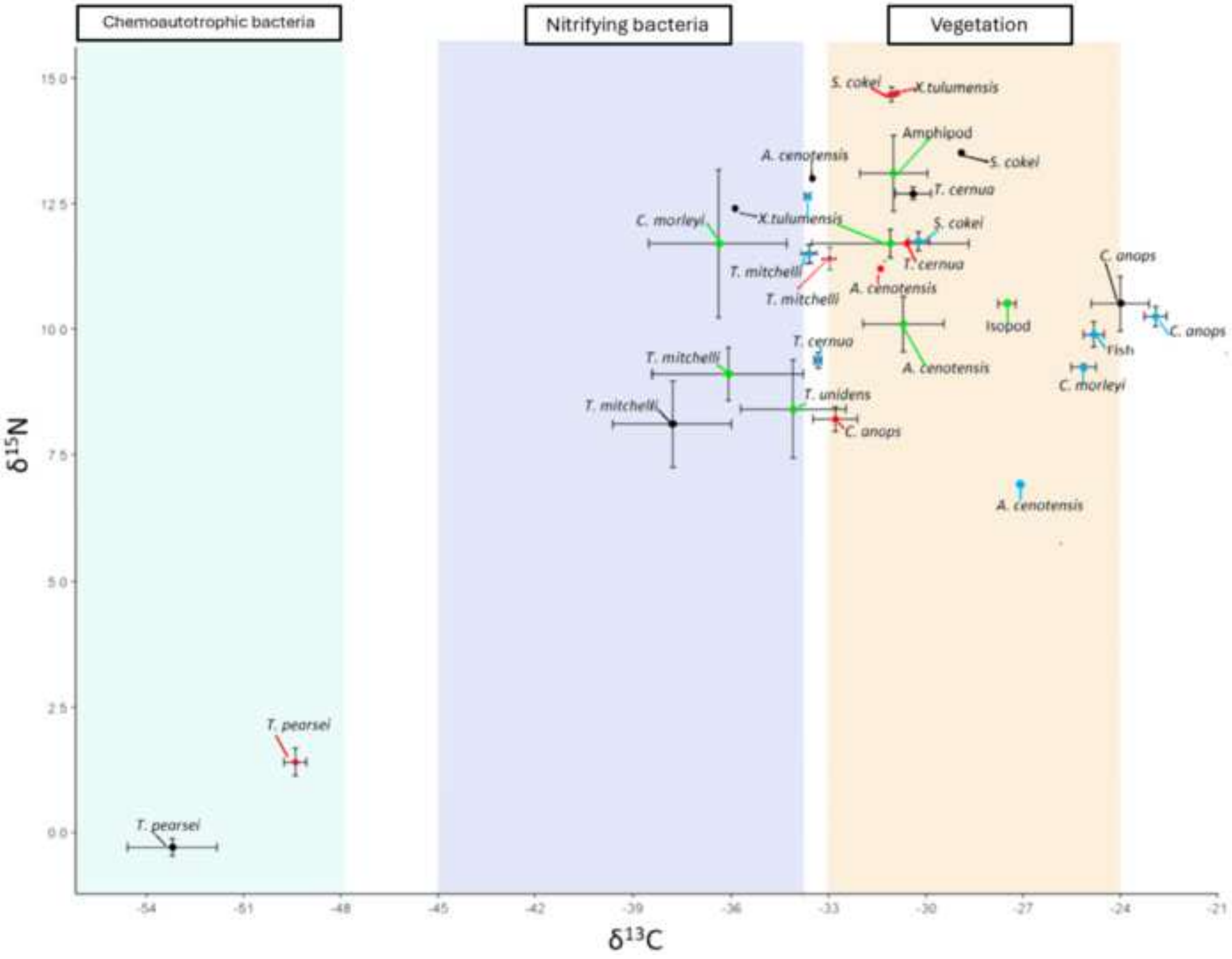

Figure 5

Click here to access/download;Figure;Fig 5.tif

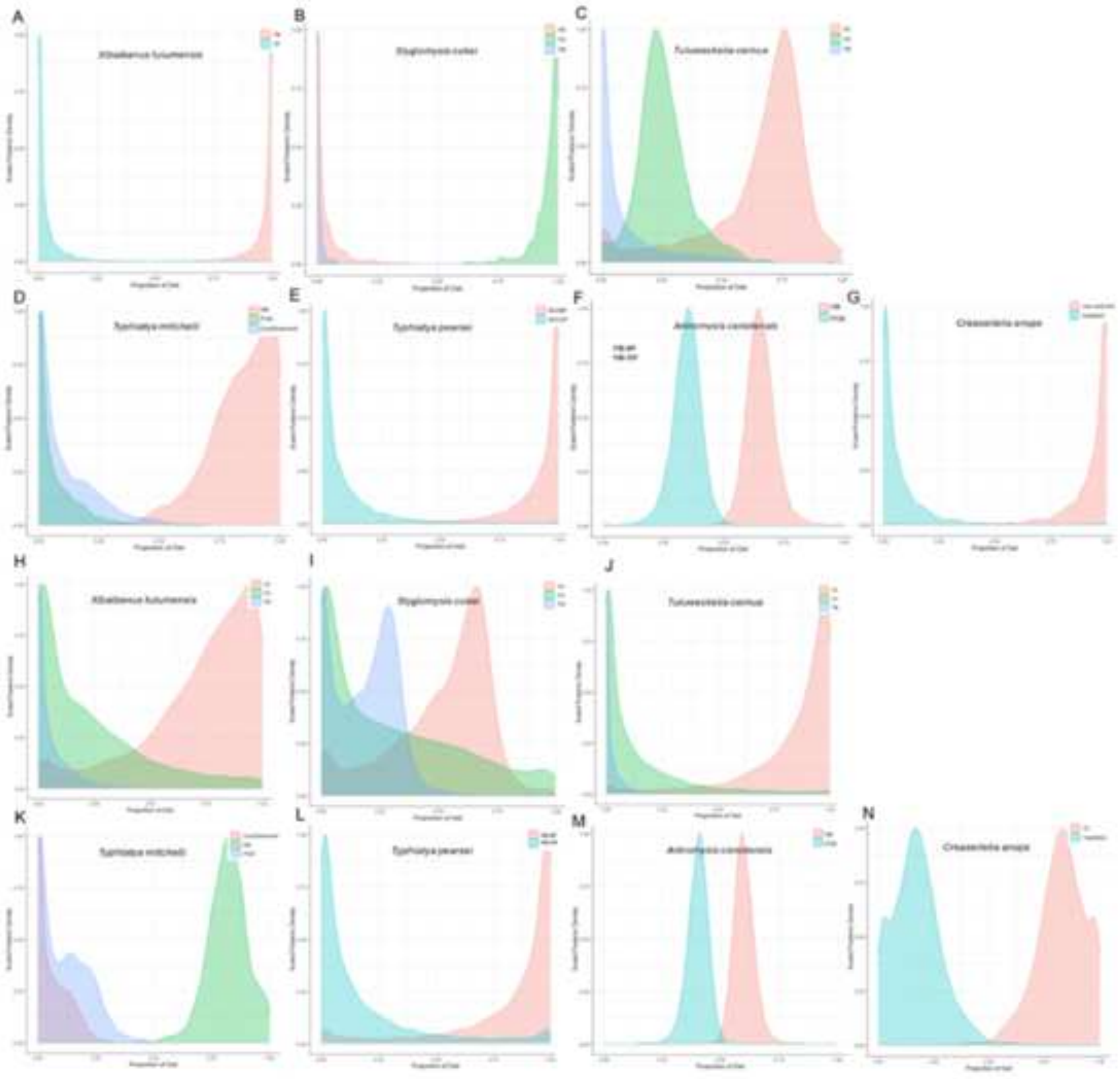

Figure 6

Click here to access/download;Figure;Fig 6.tif

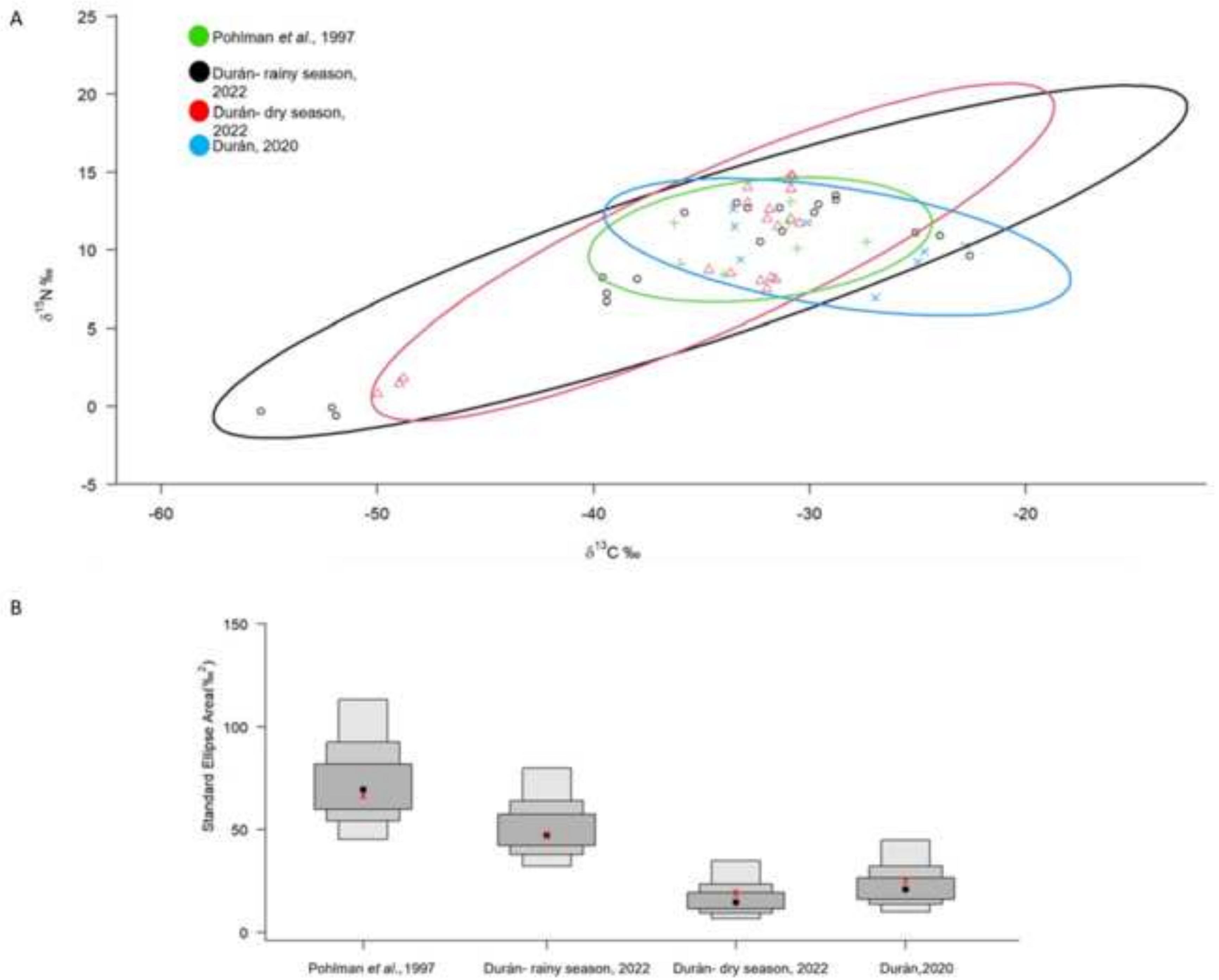

Supplement: Attachment — Submitted filename: PONE-D-24-24608_rev.pdf [file pone.0315821.s003.pdf]
