# Supplementary material for: Trophic ecology in an anchialine cave: A stable isotope study
Source: PLoS One. 2025 Jan 3;20(1):e0315821. doi: 10.1371/journal.pone.0315821 (PMC11698404; doi:10.1371/journal.pone.0315821)
Supplement: Attachment — Submitted filename: response to reviewers.docx [file pone.0315821.s004.docx]

Response to reviewers

Journal requirements:

1. We followed the style templates for “main body” and title, authors, affiliations”.
2. We have stated in the cover letter that “The funders had no role in study design, data collection and analysis, decision to publish, or preparation of the manuscript”.
3. We removed all funding-related text from the “Acknowledgements” section. We have now included the funding information in the cover letter.
4. We have uploaded two supporting information files to complete the analyzed in this study.
5. – (this was skipped in the editor’s letter).
6. An improved version of Figure 5 has been uploaded.
7. We have created a new map for Figure 1, which now contains only elements that can be used freely.

Reviewer 1 (marked copy).

Line 20. The term “unique” was changed for “endemic”.

Line 21. We added at the end of the sentence “analyzed to determine what the main nutrient sources are”, to complete the idea.

Line 23. We now specify that the seven species we studied are the same that were studied in the other two previous studies (line 33 in the new version).

Line 25. The reviewer asks what we mean by “anchialine niche”, now it reads “anchialine isotopic niche”, clarifying the idea.

Lines 25-27. Subsection “b” of this sentence now reads “. . . . confirm previous trophic classifications; . . . “. The idea is that based on morphology alone the studied species, which are endemic and rare, were assigned by different authors a hypothetical trophic role, our study contributes with the contribution models additional evidence of what the trophic role is for each one.

Line 30. The reviewer asks what is original about our findings, I will refer to lines 23-30 of the marked copy. Subsection “a”, no previous study have compared the δ^13^C and δ^15^N values of anchialine fauna through time, which is a central point in our study; “b”, as mentioned above, anchialine species are rare and their trophic roles have been inferred from anatomical attributes and a few observations at the community level, now we analyze their trophic roles through contribution models based on stable isotope data; “c”, no previous study have proposed which of the anchialine species in the YP could be the main link in a microbial based food web, including methanotrophic bacteria, and thus here we present evidence that points to *Typhlatya pearsei* as the species that has this crucial role.

Line 38. The word “visualize” has been changed for “identify”.

Line 40. The suggested reference “Mammola et al., 2021” was included in the sentence.

Line 48. The reviewer assumes that because our study site is in the Yucatan Peninsula the water in the caves have more organic resources (nutrients) compared to temperate regions. Water quality in the flooded caves of the YP has been many times considered as oligotrophic or even ultraoligotrophic in preserved areas (Álvarez et al., 2023. Anchialine Fauna of the Yucatan Peninsula: Diversity and Conservation Challenges. In: Jones, R.W., Ornelas-García, C.P., Pineda-López, R., Álvarez, F. (eds) Mexican Fauna in the Anthropocene. Springer, Cham. <https://doi.org/10.1007/978-3-031-17277-9_13>; Torres-Talamante et al., 2011, The key role of the chemolimnion in meromictic cenotes of the Yucatan Peninsula, Mexico. *Hydrobiologia* **677**, 107–127 https://doi.org/10.1007/s10750-011-0746-9). Of course, intensive development in the region has contaminated to different extents specific sections of the aquifer as it occurs around the world, but large portions of the flooded caves network remain in good condition with very low nutrient concentrations (e.g., Metcalfe et al., 2011 – Environmental Pollution 159:991-997). In any case, the evolution of the anchialine fauna has occurred in low nutrient conditions in all anchialine caves around the world (Iliffe & Alvarez, 2018 – Research in anchialine caves in O., Kováč, Ľ., Halse, S. (eds) Cave Ecology. Ecological Studies, vol 235. Springer, Cham. <https://doi.org/10.1007/978-3-319-98852-8_18>).

The reviewer also states that this cave is “less divergent from the surface environment”. We do not know on what data is the reviewer founding this assertion. Our opinion and that of many authors is that the anchialine cave environment is unique and cannot be compared to the surface environment.

Line 55. The correction has been made, we changed “different” for “differently”.

Line 57. The sentence was deleted and incorporated in the last paragraph of the Introduction.

Line 64. The reviewer suggests that the definition of “cenote” here is not necessary because it was mentioned lines above. However, since we moved the sentence (line 57) where this was mentioned, now we feel that the definition is necessary.

Line 89. The reviewer comments: “you are jumping from one topic to the other and the reading is not linear”.

Paragraph from lines 36 to 44, topic – stable isotope analysis could be useful to study the anchialine trophic web

Paragraph from lines 45 to 59, topic – anchialine caves have a rich fauna and are characterized by low nutrient concentrations.

Paragraph from lines 60 to 79, topic – previous studies of anchialine fauna in the Yucatan have described the possible trophic roles of several species.

Paragraph from lines 80 to 92, topic – SIA has identified that nutrients could also enter the anchialine environment through microbial activity.

Paragraph from lines 93 to 100, topic – from SIA data it is possible to link microbial activity to the trophic role of certain species.

Paragraph from lines 101 to 110, topic – recent studies that identify the existence of chemosynthesis in the anchialine environment support these results.

Paragraph from lines 111 to 120, topic – summary of relevant questions that we are addressing in this study.

This is the sequence of ideas that are dealt with in the introduction. We go from general to particular ideas; however, to clarify this sequence we added the following sentence at the end of the first paragraph: “In order to follow a logical sequence, we need to know what species occur in the anchialine habitat and what their likely trophic roles are, how nutrients are entering the caves and what the possible sources are, and finally under what degree of variation – spatial and temporal – is this structure operating?

Line 111. We agree with the comment. However, we deem improbable that the anchialine cave environment could suffer the invasion of “allochthonous (epigean)” species due to an increased nutrient input. Rather, the most probable effect would be the loss of native species.

Line 117. We compare the results of this study with a previous one conducted in the same cave two years before and with another one conducted 18 years ago with the same species in a nearby cave. We are aware of the shortcomings of this comparison, but this the only other comparable study available.

Line 141. We are aware of the limitations of the comparison. However, using a study conducted in the same way, with the same species, in a similar cave, only a few kilometers away, represents a good opportunity to gain knowledge about the scale of temporal variation in the trophic structure of this community.

Line 145. More detail on the sampling of organisms has been added (lines 188-189 in the new version)

Line 148. Up to 50 m around the cenote, this has been included in the text.

Line 148. There are fish in the cenote pool, mainly of the genus *Astyanax*, that eat all insects and other small animals that fall into the pool. Whereas leaves and other plant material sink and collect in the bottom undergoing a slow decaying process. During this time is when it can be transported into the cave.

Line 190. See comment number 6 under Reviewer 2.

Line 213. As is shown later in the section and in Figure 3, there is one complete water quality profile for each one of the samplings.

Line 256. The studied species are now mentioned in the last paragraph of the Introduction.

Line 260. We are just referring to Table 3, mentioning the species that have the maximum and minimum values for each isotope.

Line 284. See comment 6 of Reviewer 2 below.

Line 299. We are referring to Figure 5A, this is a result of our study.

Reviewer 2.

1. Line 173. The reviewer asks for more details on the collecting methods and sample sizes.

Vegetation samples consisted of leaves lying on the rainforest floor coming from the more common trees around the cenote. The most common species around the cenote are listed in the manuscript.

More details of how the water samples were obtained were added. Three filtered subsamples were obtained from the pool, cave freshwater and cave salt-water.

Regarding the samples for SIA, we explain that in the case of small species (mysid shrimps and amphipods) several organisms were used to obtain the required sample for the analysis. With larger species (isopods, atyid and palaemonid shrimps, remipedes) one organism was used to obtain one sample.

2. Table 1. There are no dispersion measures because there was one water sample for each section of the system.
3. Table 2. There were 7 sediment samples taken and analyzed to cover the transect from the rainforest floor to 75 m inside the cave. The idea was to determine how the organic and inorganic carbon fractions varied from the outside to inside the cave.
4. Table 3. We now specify that “n” is the number of samples analyzed, not the number of organisms. We explained in Mat & Meth that we used several organisms for one sample in case they were very small (mysid shrimps and amphipods) and that we used one organism per sample with large specimens.
5. Figure 4. We have modified the figure to improve its clarity.
6. Table 4. The reviewer questions the usefulness of the ANOVA used to test for differences in isotopic values between species and between seasons. Since the number of values is limited due to the difficult conditions to sample this environment and the terms of the ANOVA cannot be improved, we follow his/her advice and have eliminated this analysis from the manuscript. Instead, we added more detail to the direct comparison of δ^13^C values.
7. Line 298. Yes, this is a very pertinent comment by the reviewer. We have now improved the paragraph where the results of the contribution models are presented. We added the information that allows the reader to know in which cases a change in the diet composition occurs with a change in the δ ^13^C values of the species. As is now presented, in some cases the δ ^13^C values change with the diet and in others it doesn’t.
8. Figure 5. The figure has now been improved as suggested.
9. Bayesian mixing models are described in the “Data analysis” section of Materials and Methods. We expanded this explanation and added a reference.
10. The reviewer comments on the data presented in Table 6 (Table 4 in the new version), which might be confusing. The isotopic values from other sources that are used for comparisons need a more detailed explanation. We have added notes to Table 4 to improve the clarity.
11. This is an interesting question by the reviewer. The reviewer asks if *Typhlatya pearsei* is the only species that has the role of consuming methanotrophic bacteria. Our data shows that in this cave it is the only species that can have such a role. We have added a couple of sentences in the Discussion to emphasize this point (lines 492-496 of the new version).

Line 18. Correction done.

Line 80. Correction done.

Line 107. Correction done (line 126 in the new version).

Line 115. Correction done (line 134 in the new version).

Line 153. Changes done (line 185 in the new version).

Line 220. Change done (line 269 in the new version).

Line 225. Change done (line 275 in the new version).

Lines 360-365. Sentence too long. The sentence has been divided to improve clarity (lines 435-438 in the new version).

Reviewer 3.

1. Figures 3 and 5 have been modified and improved. The rest of the figures have been checked
2. We have added a paragraph in the Introduction emphasizing the nutrient-poor condition of anchialine caves in the Yucatan.

Lines 17-18. Corrections done.

Lines 80-85. The sentence has been divided as it was too long and hard to read (lines 105-111 in the new version).

Lines 108-109. The sentence was modified as suggested (lines 135-136 in the new version).

Line 120. The sentence was modified as suggested (lines 155-156 in the new version).

Lines 131-132. The sentence was modified as suggested (line 168 in the new version).

Lines 201-203. The reviewer suggests that the description of the graphs used to represent the results of the Bayesian mixing models (BMM) presented in the “Materials and methods” section should be associated to a figure. When the results of the BMM are presented they are associated to a figure (paragraph starting on line 356 of the new version). In any case, we added a call to Fig 5 in that paragraph, and a call to Fig 6 in the next paragraph.

Lines 368-369. The sentence was modified as suggested (lines 455-456 in the new version).

Line 370. Change done (line 457 in the new version).

Line 471. Correction done (line 568 in the new version).

Table 1, lines 244-245. Correction made (line 307 in the new version).

Table 2, lines 251-252. Correction made (line 315 in the new version).

Figure 6, line 343. Correction made (line 432 in the new version).
